# Operationalising the ESSENCE principles for equitable global health research partnerships in low- and middle-income countries: lessons from the RESPIRE collaboration

**DOI:** 10.7189/jogh.16.03031

**Published:** 2026-09-04

**Authors:** Hani Salim, Biswajit Paul, Siti Nurkamilla Ramdzan, Rutuja Patil, GM Monsur Habib

**Affiliations:** 1 Department of Family Medicine, Faculty of Medicine and Health Sciences, Universiti Putra Malaysia, Serdang, Malaysia; 2 Rural Unit for Health and Social Affairs, Christian Medical College, Vellore, Tamil Nadu, India; 3 Department of Primary Care Medicine, Faculty of Medicine, Universiti Malaya, Kuala Lumpur, Malaysia; 4 Vadu Rural Health Program, KEM Hospital and Research Centre, Pune, India; 5 Community Respiratory Centre, Bangladesh Primary Care Respiratory Society, Khulna, Bangladesh

**Keywords:** global health, research capacity, low- and middle-income countries

## Abstract

Despite growing calls to decolonise global health, many research capacity strengthening initiatives in low- and middle-income countries (LMICs) remain externally driven, short-term, and focused primarily on individual training rather than sustainable institutional ecosystems. Drawing on experiences from the RESPIRE collaboration across South and Southeast Asia, we reflect on key lessons and enabling mechanisms for operationalising equitable global health research partnerships through the ESSENCE. RESPIRE illustrates how capacity strengthening can move beyond transactional partnerships towards locally anchored, sustainable, and LMIC-led research ecosystems grounded in local leadership, regional peer learning, governance, mentorship, and community engagement. These experiences offer practical lessons for funders, institutions, and partnerships seeking to strengthen equitable research capacity in LMICs.

Calls to decolonise global health have intensified in recent years, highlighting persistent inequities in agenda-setting, authorship, funding control, and institutional leadership within global health research partnerships [1]. Despite increasing recognition of these inequities, many partnerships continue to operate through funding, governance, and publication structures that privilege high-income country (HIC) institutions. Research capacity strengthening initiatives in low- and middle-income countries (LMICs) often remain short-term, externally driven, and focused primarily on individual training rather than sustainable institutional ecosystems.

These challenges are particularly relevant in respiratory health. Respiratory diseases, including tuberculosis, pneumonia, asthma, and chronic obstructive pulmonary disease, remain major contributors to global morbidity and mortality [2]. Yet respiratory health has historically received less attention within global health funding and research agendas than other major communicable and non-communicable diseases [3]. In response to these gaps, the National Institute for Health and Care Research Global Health Research Unit on Respiratory Health (RESPIRE), established in 2017, adopted a collaborative model to strengthen research and health systems capacity across South and Southeast Asia.

Using the Seven Principles for Strengthening Research Capacity (ESSENCE) as a guiding framework [4], we reflect critically on the experiences of the RESPIRE collaboration and argue that sustainable research capacity strengthening requires moving beyond transactional north–south partnerships towards long-term ecosystem approaches grounded in equity, local ownership, regional collaboration, and shared leadership.

## PRINCIPLE 1: NETWORK, COLLABORATE, COMMUNICATE AND SHARE EXPERIENCE

RESPIRE was founded on collaboration among LMIC institutions. Initially operating in Bangladesh, India, Malaysia, and Pakistan, it later expanded to Bhutan, Indonesia, and Sri Lanka. Unlike traditional north–south collaborations centred primarily on bilateral HIC–LMIC relationships, RESPIRE fostered regional peer learning across LMIC partners through multicountry studies [5–7], annual scientific meetings, thematic working groups, and cross-site exchanges. These platforms enabled partners to share implementation experiences, troubleshoot context-specific challenges, and build professional networks that extended beyond individual projects.

## PRINCIPLE 2: UNDERSTAND THE LOCAL CONTEXT AND ACCURATELY EVALUATE EXISTING RESEARCH CAPACITY

Global health interventions may be unsuccessful when they are insufficiently adapted to local health systems, sociocultural realities, and resource constraints. RESPIRE embedded contextual understanding, local needs assessments, and stakeholder engagement into research design and capacity strengthening activities. For example, digital auscultation research in Bangladesh responded to diagnostic challenges in resource-constrained clinics, while educational and community-based respiratory interventions in India and Bangladesh were developed with healthcare providers and communities to improve feasibility and acceptability [8–11].

These locally grounded approaches reinforced the importance of context-sensitive implementation research. They also revealed challenges between local flexibility and the standardisation often required in multicountry programmes. RESPIRE’s experience suggests that equitable global health research requires adaptation rather than direct replication of interventions across settings.

## PRINCIPLE 3: ENSURE LOCAL OWNERSHIP AND SECURE ACTIVE SUPPORT

From its inception, RESPIRE involved LMIC partners in identifying research priorities, designing studies, leading implementation and adapting interventions to local health-system needs. Strategic programme leadership was shared through a Unit Management Committee representing all partner countries, enabling collective decisions on research priorities, programme direction and major operational matters. At each RESPIRE site, LMIC investigators led local teams and managed relationships with communities, healthcare providers and policymakers. Within agreed study protocols, they made decisions about how recruitment, intervention delivery and stakeholder engagement should be adapted to the local context. For multicountry studies, common protocols and cross-site issues were discussed through thematic working groups and regular meetings involving site and programme leads. These arrangements enabled LMIC researchers to progress from contributing to studies to leading projects, publications and regional collaborations. However, their autonomy remained bounded by an externally funded structure in which overarching budgets, contracts and reporting requirements were substantially shaped by the UK funder and coordinating institution.

The development of the Pulmonary Rehabilitation initiative across Bangladesh, India, and Malaysia illustrates how locally led projects can evolve into wider multicountry collaborations [7,12]. Initially developed through Dr GM Monsur Habib’s RESPIRE PhD dissertation, the programme expanded as other centres recognised its relevance and adapted it within their own settings.

RESPIRE also embedded participatory approaches involving patients, policymakers, and healthcare providers, including Photovoice and school-based asthma initiatives that foregrounded community perspectives and strengthened research relevance [5,6].

## PRINCIPLE 4: BUILD IN MONITORING, EVALUATION AND LEARNING FROM THE START

Structured monitoring, evaluation, and regular reflection were integral to RESPIRE. Capacity outcomes and implementation experiences were reviewed throughout the programme, allowing training, mentorship, and implementation strategies to be adapted over time. Beyond traditional academic outputs, RESPIRE emphasised broader indicators of capacity strengthening, including stakeholder engagement, leadership development, implementation experience, and policy influence [5,13].

Cross-cutting platforms in community engagement and involvement, education and training, data sharing and open science, knowledge mobilisation, and digital innovation supported collaboration and learning across partner institutions. However, RESPIRE also illustrates a wider challenge in research capacity strengthening: outcomes such as leadership growth, institutional culture change, and sustained regional collaboration are difficult to capture using conventional academic metrics alone.

## PRINCIPLE 5: ESTABLISH ROBUST RESEARCH GOVERNANCE, SUPPORT STRUCTURES AND PROMOTE ACTIVE LEADERSHIP

RESPIRE invested in research administration, ethics, and data governance across partner institutions. Training in research governance, standard operating procedures, and Good Financial and Grant Practices certification strengthened institutional readiness and administrative capacity. Beyond technical systems, RESPIRE supported leadership development through multicountry mentorship, regional collaboration, and exposure to global research networks. Early-career researchers were supported to lead national and international initiatives [14], contribute to policy discussions, and adapt global evidence to local contexts. This participatory leadership approach helped decentralise expertise and strengthen institutional ownership within LMIC settings.

## PRINCIPLE 6: EMBED STRONG SUPPORT, SUPERVISION AND MENTORSHIP STRUCTURES

RESPIRE embedded mentorship through co-supervision, peer networks, and support for doctoral and postdoctoral researchers. Twelve PhD scholars joined the programme in 2018, each undertaking locally relevant respiratory health research. Mentorship combined scientific rigour with contextual understanding and encouraged reciprocal learning between LMIC and HIC researchers [15]. Many scholars have since moved into leadership roles, mentoring junior researchers, contributing to policy, and sustaining local research communities.

Reflecting on his experience in Bangladesh, a scholar shared:

RESPIRE brought research into my clinical practice for the first time. Until then, I searched for answers in global literature that often lacked relevance to our setting. Through my PhD, I came to see that research is not separate from clinical care - it is essential to improving it.

Similarly, another scholar described how RESPIRE strengthened her independence, leadership, and advocacy work for children with asthma in Malaysia. These reflections illustrate how locally relevant research and supportive mentorship can transform clinicians into clinician-researchers. They also show that capacity strengthening involves not only technical training, but also confidence, professional identity, peer support, and sustained research communities.

## PRINCIPLE 7: THINK LONG-TERM, BE FLEXIBLE AND PLAN FOR CONTINUITY

RESPIRE’s capacity strengthening approach extended beyond individual projects and short-term outputs. The programme aimed to build institutional and regional capacity through policy engagement, digital health infrastructure, cross-institutional supervision, and long-term collaboration. Its regional model aligns with broader calls to decolonise global health and shift leadership towards the Global South [1]. Future partnerships must therefore invest not only in research projects, but also in enduring institutional systems, regional networks, and leadership pipelines.

## LESSONS LEARNED FROM RESPIRE IMPLEMENTATION

RESPIRE provides practical insights into research capacity strengthening in LMICs using the ESSENCE principles. **Table 1** summarises the main enabling mechanisms and challenges identified through its implementation. These lessons should, however, be interpreted in light of the enabling conditions and structural constraints that shaped RESPIRE.

**Table 1:** Lessons from RESPIRE on research capacity strengthening in LMICs

What worked well	
Local ownership and leadership	Delegating operational decisions to site partners strengthened local ownership and supported sustainability; early engagement with local governments and stakeholders supported planning for continuity.
Cross-country collaboration and peer learning	Regular engagement through annual meetings and thematic groups strengthened regional partnerships and knowledge exchange, fostering mutual learning and collective problem-solving.
Structured mentorship and supervision	The strong emphasis on mentorship and co-supervision enabled early-career researchers to gain rigorous academic training while addressing local priorities. The continued engagement of PhD graduates in leadership roles validated the effectiveness of this supportive structure.
Embedded feedback mechanisms	Integrating structured monitoring and evaluation from the outset allowed iterative refinement of capacity-building activities, ensuring adaptability and relevance over time.
**Challenges and areas for improvement**	
Ensuring sustainable funding	While RESPIRE successfully demonstrated scalability and local adoption, sustaining research ecosystems beyond externally funded programme cycles remains one of the greatest unresolved challenges in LMIC capacity strengthening.
Capacity building in research governance	While considerable strides were made, further investment in administrative and regulatory capacity is necessary to ensure that local institutions can independently manage complex research governance and ethics oversight in the long term.
Inclusivity and diversity:	Despite significant advances, ongoing efforts are needed to ensure diverse representation, especially gender equity, at all stages of research. Ensuring equitable opportunities and active participation requires continuous intentionality and monitoring.

LMIC – low- and middle-income countries, RESPIRE – REspiratory Science Promoted by International Research Exchanges

RESPIRE benefited from long-term funding, established partner institutions, doctoral training and sustained opportunities for cross-country engagement. Its model may not be directly transferable in settings with shorter funding cycles, less-developed research infrastructure, political instability or limited institutional support. Nevertheless, several core practices are potentially transferable, including shared priority-setting, locally led adaptation, regional peer learning, distributed mentorship and co-design with patients and communities. These practices should be adapted to local capacity, priorities and power relationships rather than replicated without modification.

RESPIRE could not fully overcome the structural inequalities inherent in externally funded global health research. Funding flows, contracting, governance and reporting requirements remained largely determined by the funder and HIC coordinating institution. Although RESPIRE enabled LMIC partners to lead projects and publications, differences in institutional resources, administrative capacity, seniority and access to international networks continued to influence their wider participation and leadership. Distributed governance, LMIC-led implementation and regional collaboration helped reduce, but did not eliminate, these inequalities.

### Recommendations for funders and institutions

Supporting more equitable partnerships requires funders to provide longer funding periods, enable direct or jointly controlled funding for LMIC institutions, and invest in administrative, governance and research infrastructure alongside individual projects and training. Partnership agreements should establish transparent processes for decision-making, budget control, data ownership, authorship and leadership succession. Progress should be assessed using locally defined indicators of institutional capacity and equitable leadership, as well as conventional academic outputs. National and regional funders should also increase investment in locally led research to diversify funding and reduce dependence on HIC sources, particularly as international global health funding reduces.

Institutions also have important responsibilities. HIC institutions should recognise mentorship and partnership development within academic promotion systems, while LMIC institutions should provide protected research time, sustainable career pathways and opportunities for experienced researchers to mentor the next generation. Sustainability planning should begin at programme inception and include support beyond individual grants. Ultimately, long-term investment is needed to enable LMIC institutions not only to participate in international collaborations, but also to lead research that addresses their own health priorities.

## CONCLUSIONS

RESPIRE highlights the potential of collaborative, context-sensitive, and locally led approaches to research capacity strengthening in LMICs. Through its alignment with the ESSENCE principles, the programme demonstrates how equitable partnerships grounded in local leadership, regional collaboration, mentorship, robust governance, and continuous learning can support sustainable research ecosystems. Addressing under-prioritised conditions such as respiratory diseases will require long-term investment in locally anchored institutions and research networks rather than short-term project-based initiatives. While RESPIRE illustrates what is possible, sustaining these gains will require broader changes in how global health research is funded, governed, and evaluated. The experiences of RESPIRE offer practical lessons for operationalising equitable partnerships and advancing efforts to decolonise global health by enabling LMIC institutions to lead, innovate, and strengthen their own research systems.
